# Screening System of *Cannabis sativa* Extracts Based on Their Mitochondrial Safety Profile Using Cytochrome c Oxidase Activity as a Biomarker

**DOI:** 10.3390/ijms24021315

**Published:** 2023-01-10

**Authors:** Ekaterina Noskova, Roberto Fernández, Javier García, Eneko Ochoa, Celtia Domínguez-Fernández, Albert Adell, Antonio Laso, Maria Fe Andrés, Azucena González-Coloma, Egoitz Astigarraga, Gabriel Barreda-Gómez

**Affiliations:** 1 Research and Development Division, IMG Pharma Biotech, 48160 Derio, Spain; 2 Instituto de Biomedicina y Biotecnología de Cantabria, IBBTEC, Consejo Superior de Investigaciones Centíficas (CSIC), University of Cantabria, 39011 Santander, Spain; 3 Institute of Agricultural Sciences (ICA), Spanish Research Council (CSIC), 28006 Madrid, Spain; 4 Research and Development Division, AleoVitro, 48160 Derio, Spain; 5 Department of Pharmacology, Faculty of Medicine and Nursing, University of the Basque Country, UPV/EHU, 48940 Leioa, Spain

**Keywords:** cannabinoids, *Cannabis sativa* extracts, THC, CBD, cytochrome c oxidase, mitochondrial activity

## Abstract

The development of *Cannabis sativa* strains with high cannabidiol (CBD) and low tetrahydrocannabinol (THC) content is a growing field of research, both for medical and recreational use. However, the mechanisms behind clinical actions of cannabinoids are still under investigation, although there is growing evidence that mitochondria play an important role in many of them. Numerous studies have described that cannabinoids modulate mitochondrial activity both through activation of mitochondrial cannabinoid receptors and through direct action on other proteins such as mitochondrial complexes involved in cellular respiration. Thus, the aim of this study was to determine the actions of a panel of extracts, isolated from high-CBD varieties of *Cannabis sativa*, on the activity of the mitochondrial electron transport chain complex IV, cytochrome c oxidase (CCO), in order to select those with a safer profile. After demonstrating that *Cannabis sativa* strains could be identified by cannabinoids content, concentration–response curves were performed with a collection of extracts from strains with high-CBD and low-THC content using bovine CCO. The CCO rate was clearly modified by specific extracts of *Cannabis sativa* plants compared to others. Half maximal inhibitory concentrations (IC50) of extracts and the inhibitory effects evoked at 1 × 10^−4^ g/mL displayed a significant correlation with the THC. Therefore, the screening of extracts based on CCO activity provides a powerful and rapid methodology to identify those plants with higher mitochondrial toxicity or even mito-protective actions.

## 1. Introduction

*Cannabis sativa* is a natural source of cannabinoids that contains more than 500 compounds, including more than 100 phytocannabinoids that have not been identified in any other plant. Several phenotypes (chemotypes) of *C. sativa* which differ in chemical composition and ratios of active compounds were described [1,2]. The most abundant and thoroughly investigated are Δ9-tetrahydrocannabinol (Δ9-THC), non-psychoactive cannabidiol (CBD) and cannabinol (CBN) and their metabolic precursors cannabinolic acid (CBDA), and Δ9-tetrahydrocannabinolic acid (Δ9-THCA). These compounds have been studied over the last years because of their multiple pharmacological effects, such as antioxidative [3] and neuroprotective [4] actions, and anxiolytic [5] and antidepressant-like effects [6,7]. Currently, some cannabis-based medicines are legislated for therapeutic use in the USA, Europe, and Israel. For instance, Epidolex, the first cannabis-derived drug approved by the FDA for patients suffering from rare forms of epilepsy [8]. Nabiximols (Sativex), an extract consisting of THC and CBD combination, was approved for multiple sclerosis symptoms treatment [9]. Dronabinol, also known as delta-9-tetrahydrocannabinol, and its synthetic derivative, nabilone, are used in the treatment of nausea and vomiting in patients under chemotherapies [10]. All these medicines together with medical cannabis have demonstrated analgesic efficacy in chronic neuropathic pain treatment [11]. In addition, an increasing number of cannabinoids and their combinations seem promising as anti-inflammatory drugs [12], in cancer therapy [13,14], migraine [15], opioid and alcohol dependence treatment [16]. It has been shown that very low doses of THC + CBD and THCA + CBDA combinations have an anti-emetic effect [17] which is an example of a combination use advantage when compared to the administration of high doses of a single active component.

One of the mechanisms underlying the neuroprotective and antioxidative effect is cannabinoid-induced changes in mitochondrial activity. It has been demonstrated that these molecules act through their specific G protein-coupled receptors, CB_1_ and CB_2_, which mainly are located at the plasmatic membrane. However, around 30% of the neuronal mitochondrias also present CB_1_ in their external membrane, revealing a crucial role of these receptors in mitochondrial function [18]. In this sense, recent studies demonstrate that mitochondrial cannabinoid receptors can modulate learning processes by the control of the mitochondrial electron transport chain (mETC) via a signal pathway that involves Gαi protein, soluble-adenylyl cyclase (sAC), and protein kinase A (PKA) [19]. On the other hand, there are evidence of a non-receptor mechanism involved in the cannabinoid modulation of oxidative phosphorylation (OXPHOS) [20,21,22]. The main purpose of this process is the ATP formation using the energy of the electrochemical gradient; however, during the mETC functioning, the reactive oxygen species (ROS), such as hydrogen peroxide (H_2_O_2_), superoxide anion (O^2−^), and hydroxyl radical (HO•), are also forming. The imbalance between ROS overproduction and the function of endogenous antioxidants may cause oxidative stress associated with cell death and, as a result, organ systems damage [23]. Moreover, ROS accumulation is implicated in many neurodegenerative diseases, including Alzheimer’s [24] and Parkinson’s [25] disease and other pathology states such as inflammation, fibrosis, and cancer [23].

The effect of cannabinoids on ROS formation is mentioned in some studies regarded to cancer [14], neurodegenerative diseases [26], and diabetes [27,28]; however, the data seems to be incomplete and controversial for some tissue types. Thus, it has been found that cannabidiol enhances ROS production in cancer cells, and human glioma cells [29], but reduces it in pancreatic islet cells [28]. It has been suggested that an increase in the production of ROS in microglial cells is provoked by CBD interaction with voltage-dependent mitochondrial membrane channels and the enhancement of intracellular calcium levels [14]. Nevertheless, the mechanisms involved in the effect of these compounds on ROS formation are still unclear.

**Figure 1:**
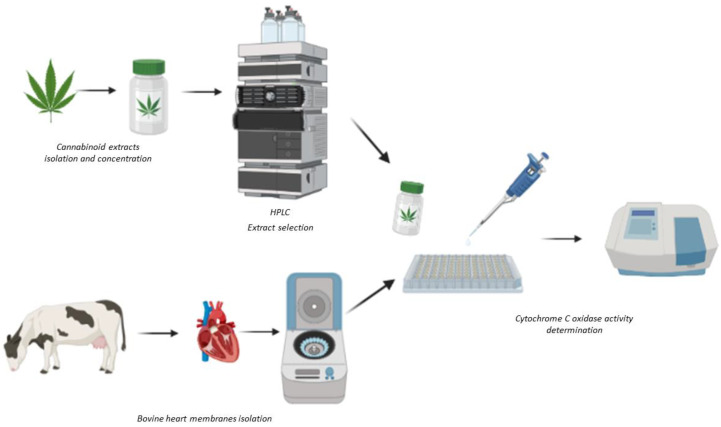
Scheme of the methodology used for sample preparation and the determination of cytochrome c oxidase activity. Created with BioRender.com.

Since cannabinoids can interact directly with mitochondrial electron transport chain complexes; the aim of this study was to develop a screening method using bovine membranes containing functional mETCs to study the actions of a panel of extracts, isolated from in vitro cloned high CBD-containing strains of *Cannabis sativa*, on the activity of mETC complex IV, cytochrome c oxidase (CCO) (Figure 1).

## 2. Results

### 2.1. Chemical Analysis of Plant Varieties

The results of the chemical analysis are shown as the mean values of CBD, CBDA, THC, THCA for each variety (Table 1, and for the complete set of data see Table S1). The plant samples showed similar concentrations of the target compounds analyzed (CBD, CBDA, THC, THCA). Some quantitative variations were observed between plant varieties, with P0 having the highest CBD and A7, D2, and T4 the highest THC content. The chromatogram of the standards’ mixture and the chromatogram of a representative sample are shown in Figure S1.

**Figure 2:**
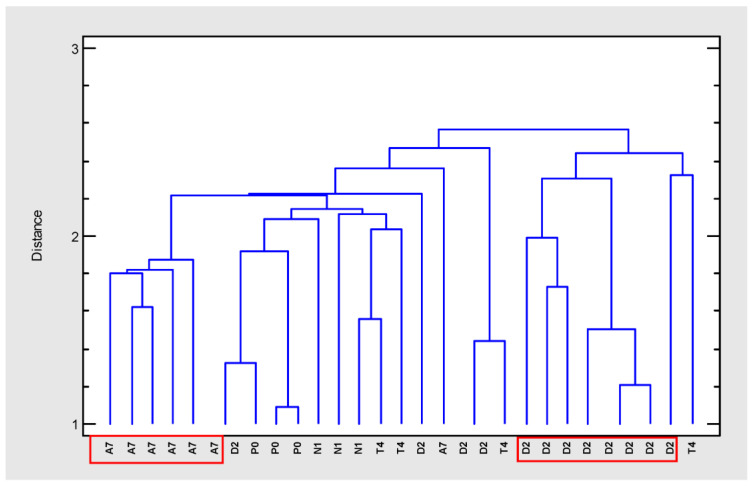
Dendrogram (farthest neighbor method, housing block) generated from cluster analysis of GC–MS data of extracts from five *Cannabis sativa* varieties (A7, D2, N1, P0, T4).

In order to group the cloned plant samples according to their composition, a cluster analysis was carried out (cluster method: nearest neighbor, distance metric: housing block) (Figure 2). Four separated groups could be distinguished at an arbitrary distance of 2.5. Two separated and homogeneous groups included the clones from varieties A7 and D2 while the other two groups were more heterogeneous. Therefore, the most differentiated and homogeneous groups were selected for further tests (A7 and D2).

### 2.2. Classification of Plant Varieties

**Figure 3:**
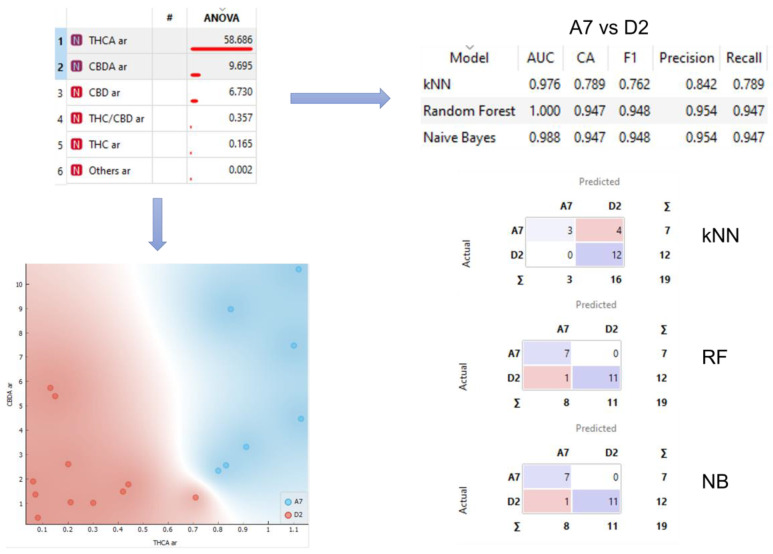
Analysis workflow. Sample separation for selected variables and confusion matrices for kNN, RF, and NB models.

Some of the variables, mainly THCA and, not so clearly, CBDA, seem to be able, individually, to distinguish between the two different varieties that have a greater number of samples, A7 and D2, as can be seen in the graph shown in the bottom left corner of Figure 3. There are not enough samples to properly check that ability with the rest of varieties.

Looking for a way to improve the individual ability of each variable to classify the varieties, we decided to use both, at the same time, to train three supervised models, naive Bayes (NB), random forest (RF), and k-nearest neighbors (kNN). Therefore, the models were trained using THCA and CBDA data of the varieties A7 and D2. In the upper right corner of Figure 3, the results of the 10-fold cross validation of the models are shown, reaching a precision of up to 0.954 for NB and RF. In the bottom right corner of Figure 3 the confusion matrixes of the three models are shown.

### 2.3. Cytochrome c Oxidase Activity Assay

To characterize the cannabinoid extracts’ effect on the CCO activity, the DAB oxidation in the presence of cytochrome c (the electron transporter between mitochondrial complexes III and IV) was studied using the methodology described in Section 4. After several trial tests, the optimal rank of concentrations was established. The effect of serial dilutions from 1 × 10^−^^6^ g/mL to 1 × 10^−^^4^ g/mL of the extract was evaluated in this study. At an extract concentration exceeding 1 × 10^−^^4^ g/mL, saturation was observed.

**Figure 4:**
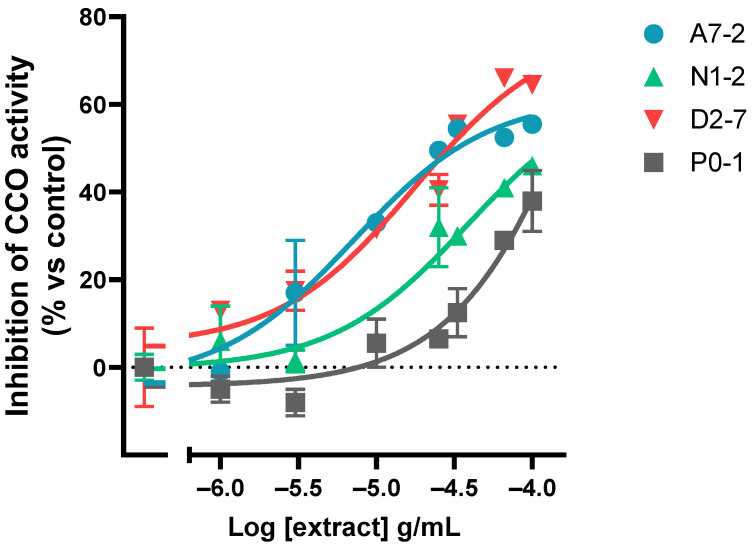
Representative graph of the cannabinoid extracts’ dose-dependent effect on the CCO activity expressed as a percentage over the basal activity in the presence of vehicle. Data are mean ± SEM values.

DAB oxidation curves were obtained for each concentration of each extract to establish the oxidation rate within the linear range (Figure S2). The assay results showed that all of the extracts tested inhibited the activity of the mitochondrial complex IV in a dose-dependent manner. The rate of inhibition of CCO was presented as a percentage relative to the control. Afterward, the logarithm of half-minimum of inhibitory or effective concentration (pIC50 or pEC50) was calculated. A model of log(agonist) vs. response (three parameters) was used for all the extracts (Figure 4 and Figure S3).

The assay results showed that different samples inhibited the CCO activity to varying degrees when applied at the same concentration: the percentage of 1 × 10^−^^4^ g/mL concentration effect ranged from 33.0% to 64.5%. The maximum variability between the percentage of CCO inhibition was observed at 1 × 10^−^^4.5^ g/mL (it ranged from −12.5% to 56%). In relation to the potency, it was found that the range of pIC50 was from −5.15 to −4.01 (Table S1). The sample with a major potency was A7-2 and the sample with the least potency was D2-10.

**Figure 5:**
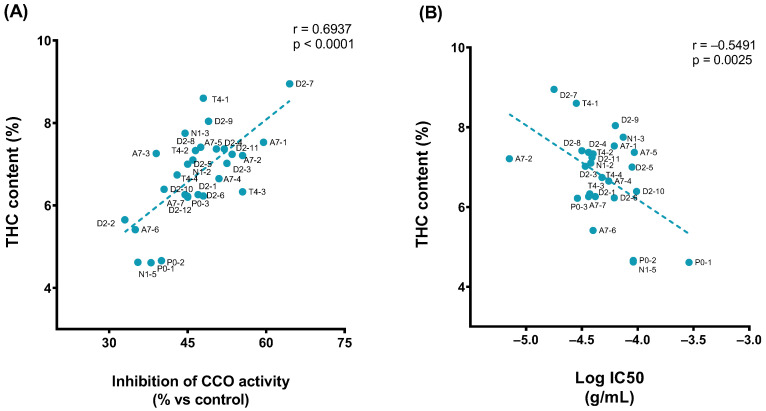
Pearson correlation plot of the (**A**) THC content (% relative area) vs. inhibition effect of 1 × 10^−^^4^ g/mL of extract (%). (**B**) THC content (% relative area) vs. pIC50.

Moreover, it was found that the effects of 1 × 10^−^^4^ g/mL of extracts significantly correlated with THC content in the samples (Figure 5A) as well as the potency of extracts (Figure 5B).

**Figure 6:**
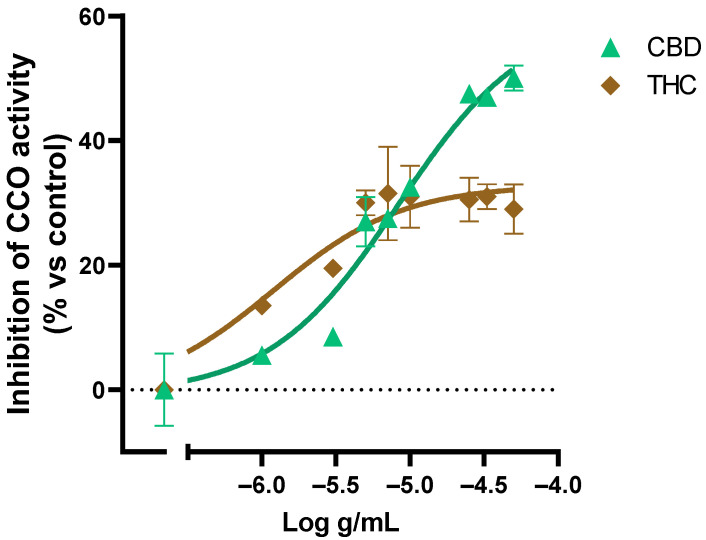
Effect of CBD and THC on the CCO activity expressed as a percentage over the basal activity in the presence of vehicle. Data are mean ± SEM values.

We also evaluated the effect of pure THC and CBD on the CCO activity (Figure 6). The results showed a saturation of the inhibitory activity of CBD in concentrations above 1 × 10^−4.6^ g/mL and THC above 1 × 10^−5.3^ g/mL, which is consistent with the *Cannabis sativa* extracts´ effect on the CCO activity where the saturation at 1 × 10^−4^ g/mL of extract was observed.

### 2.4. Superoxide Formation Assay

**Figure 7:**
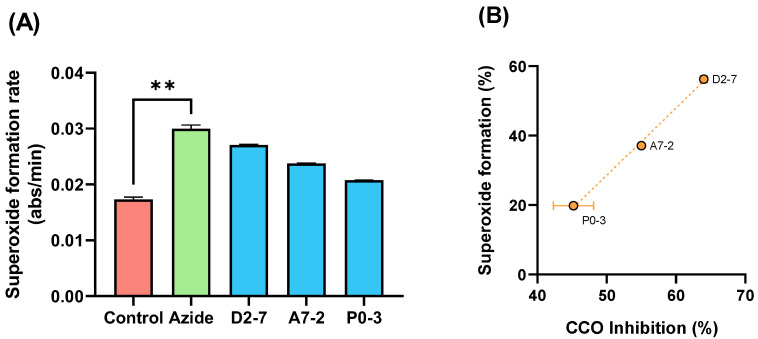
Diagram of the superoxide formation mediated by sodium azide and samples D2-7, A7-2, P0-3, chosen by THC content (** *p* < 0.01) (**A**) Correlation between the superoxide formation and (**B**) CCO inhibition mediated by extracts D2-7, A7-2, P0-3. Both expressed as percentage vs. control. Data are mean ± SEM.

To confirm the effect of the extracts on the CCO activity, we evaluated the superoxide formation mediated by samples D2-7, A7-2, and P0-3. We used succinate as a substrate for mETC functioning and compared the superoxide formation in the presence or absence of sodium azide (a CCO inhibitor). The results showed a significant increase in the condition of azide in comparison with basal activity of superoxide formation (Figure 7A). An extracts-mediated increase in the superoxide formation positively correlated (*p* = 0.0316 by Pearson correlation test) with CCO inhibition was also obtained (Figure 7B).

## 3. Discussion

Cannabidiol and tetrahydrocannabinol are cannabinoid compounds that have demonstrated analgesic efficacy in chronic neuropathic pain treatment [11], and their combinations with other drugs also suggest a promising role as anti-inflammatories [12] or as drugs for cancer therapy [13,14], migraine [15], opioid and alcohol dependence [16]. The psychoactive effects of THC have been a major limitation to the cannabinoids’ medicinal use, but the emergence of new varieties of plants with high-CBD and low-THC content opens a new window of opportunities for their use in many countries. In this sense, we have demonstrated that the concentration of certain cannabinoid compounds such as THCA, CBDA, THC and CBD is specific for each high-CBD variety of *Cannabis sativa* studied and can be used to classify two of them with a high accuracy using machine learning algorithms. Therefore, it is important to secure plant varieties with homogeneous content in target active cannabinoids by in vitro multiplication of clones. In this work, two of the five varieties cloned in vitro were separated into homogeneous groups (A7 and D2), while the rest were more heterogeneous, pointing out at the importance of variety selection for the production of chemically homogenous clones. THCA was the variable with the highest discriminatory power among the varieties, followed by CBDA, CBD, and THC. Therefore, the capacity to generate, accumulate and metabolize these metabolic precursors, THCA and CBDA, appears to be a specific characteristic of the strains examined. However, further analysis would be necessary to check whether it can be extended to other varieties.

By contrast, the screening system for *Cannabis sativa* selection, based on the actions evoked on the activity of mitochondrial respiratory complex IV, provides an easier and cost-effective method to select strains or even plants with a higher safety profile, and with less potential to generate side effects related to mitochondrial respiration. In this sense, the concentration-response curves determined in this study on bovine cytochrome c oxidase activity indicate that the THC concentration is significantly correlated with the inhibition experimented by this respiratory complex. Moreover, not only the inhibitory action of the highest concentration of the extracts displayed a significant correlation but also the half maximal inhibitory concentration determined for each extract. Furthermore, the superoxide formation evoked by activation of the mitochondrial respiration with succinate in presence of selected extracts correlates with the inhibition of the cytochrome c activity triggered by those extracts. However, no significant correlations were observed neither with CBD nor THCA or CBDA. Cannabinoid modulation of oxidative phosphorylation by a non-receptor mechanism has been described in several studies [20,21,22]. In this sense, THC and CBD can inhibit the complex IV of pig brain mitochondria [21]. According to this study, THC and CBD standards inhibited the cytochrome c activity in bovine heart membranes with similar concentration response curves. This discrepancy between the effect observed with pure cannabinoid standards and correlation studies with complex samples has also been reported in *Cannabis sativa* strains with high THC content, and could be due to other molecules, such as terpinene [30]. Indeed, it has been shown that the combination of THC and CBD is more effective for multiple sclerosis treatment [31] and pain relief [32]. Regarding the action on cellular energy metabolism and mitochondrial chain complexes, the synergetic effect of THC and CBD was also described [33]. Moreover, the potentiation of the biological effect of a compound by inactive compounds in combination is inherent in such compounds of *Cannabis sativa* as terpenoids [34].

Direct actions of cannabinoids on respiratory complexes have been described [35] independently of their action on mitochondrial cannabinoid receptors [22]. In particular, several studies have reported specific actions of THC and CBD on mitochondrial respiratory chain activity, ROS production and cellular integrity, promoting toxicological effects in certain cells that may be potentially relevant for clinical populations [36]. Furthermore, an increasing number of experimental evidences link cannabinoid modulation of mitochondrial metabolism to specific physiological processes, such as cognition and memory [19], underlining the need for accurate characterization of extracts to obtain the strains of greatest therapeutic interest.

As a response to the need for characterization of new varieties of cannabis as a promising treatment for various diseases, in this study we classified 29 *Cannabis sativa* extracts, by concentration of THCA and CBDA, and evaluated their effect over CCO activity. We have confirmed an inhibitory effect of all of the extracts tested, as well as pure THC and CBD. In contrast to pure THC and CBD, plant extracts contain cannabinoid precursors, such as CBDA and THCA, as well as other plant products that might have different effects over mitochondrial respiratory complexes such as CCO. The screening of extracts based on CCO activity provides a powerful and rapid methodology to identify those plants with higher mitochondrial toxicity or even mito-protective actions.

## 4. Materials and Methods

### 4.1. Tissue Samples and Reagents

#### 4.1.1. Drugs and Reagents

Diaminobenzidine (DAB), cytochrome c from equine heart, nitro blue tetrazolium (NBT), sodium succinate dibasic, and sodium azide were purchased from Sigma Aldrich (St-Louis, IL, USA). CBD, CBDA, CBN and THCA standards were purchased from Sigma Aldrich (1 mL of a 1 mg/mL MeOH solution, analytical standard, for drug analysis) and THC from SUPELCO (1 mL of a 1 mg/mL MeOH solution, analytical standard, for drug analysis).

#### 4.1.2. Bovine Samples

Heart samples from Bos taurus were supplied by Llodio municipal slaughterhouse (Llodio, Alava, Spain). The samples were stored at −80 °C until the membrane extraction.

### 4.2. Plant Material, Extraction and Analysis

#### 4.2.1. Plant Cultivation

Propagation from axillary or terminal buds was selected as the method for tissue culture or in vitro propagation of different chemotypes of *Cannabis sativa* L. specimens. In order to ensure genetic and chemist stability during in vitro propagation, direct organogenesis was selected as the best micropropagation technique, by regeneration from existing meristems and the formation of new shoots without an intervening callus phase.

Different combinations of modified Murashige and Skoog medium (MS), with and without plant growth regulators (PGRs), were selected and used to propagate different chemotypes of *Cannabis sativa* L. specimens. Shoots regenerated in vitro were rooted on modified Murashige and Skoog medium supplemented with 1 mg of l-indole-3-butyric acid. All the cultures were grown under controlled conditions at 25 °C ± 1 °C. The photoperiod consisted of 16 h of light and 8 h of dark. Light was provided by white fluorescent tubes of 18 W and provided and 60 ± 5 µmol m^−2^ s ^−1^ light intensity. Finally, the culture medium was replaced every 4–5 weeks.

All plants employed in this research study were grown under license for the cultivation of *C. sativa* for research purposes, issued by the Spanish Ministry of Health, Social Services and Equality via the Spanish Agency of Medicines and Health Products (Agencia Española de Medicamentos y Productos Sanitarios or AEMPS) to ALEOVITRO Ltd.

#### 4.2.2. Extraction

The plants were allowed to flower under the environmental conditions described, collected and dried at 10–15% humidity. Then, the inflorescences were subjected to Soxhlet extraction with ethanol. The solvent was eliminated in vacuo to give the extracts with yields between 32–21% (plant samples and yields are listed in Table 2 and for the complete data set, see Table S2).

#### 4.2.3. HPLC Analysis

The extracts were analyzed by HPLC-PDA in a Shimadzu unit equiped with an LC-20AD pump and a CTO-10AS VP column oven coupled to an SPD-M20A Diode Array detector. An ACE 3 C18 column (150 mm × 4.6 mm, 3 µm particle size) with an ACE3 C18 analytical pre-column was used for separation. The elution was carried out with the following gradient of acid MeOH (with 0.1% acetic acid) and acid MiliQ water (with 0.1% acetic acid): MeOH:water 80:20% during 15 min, MeOH 100% during 5 min, and MeOH:water 80:20% during 10 min at a flow rate of 0.5 mL/min. The results were analyzed at a UV wavelength of 210 nm. The stock solutions of the ethanolic extracts were injected at 1 mg/mL in 10 µL injection through an automatic injector (SIL-20A XR). All extracts were dissolved in 100% grade HPLC MeOH for injection. The identification of the products has been carried out by comparison with the retention time and UV spectrum of commercial standards.

### 4.3. Membrane Extraction

Samples were homogenized using a disperser (Ultra-Turrax^®^ T10 basic, IKA, Staufen, Germany) in 20 volumes of homogenized buffer (1 mM EGTA, 3 mM MgCl_2_, and 50 mM Tris-HCl, pH 7.4) supplemented with 250 mM sucrose. The crude homogenate was subjected to a 1500× *g* centrifugation (Microfuge^®^ 22R centrifuge, Beckman Coulter, Brea, CA, USA) for 5 min at 4 °C, and the resultant supernatant was centrifuged at 18,000× *g* for 15 min (4 °C). The pellet was washed in 20 volumes of homogenized buffer and re-centrifuged under the same conditions. Subsequently, the tubes were decanted and the pellets were frozen at −80 °C, except for one aliquot, which was used to determine the protein concentration. Protein concentration was measured by the Bradford method [37] and adjusted to the required concentrations. The heart membranes were homogenized in phosphate buffer (50 mM; pH = 7.4) and deposited in microplates using a printing solution [38,39]. 

### 4.4. Cytochrome c Oxidase Activity Determination

For the cytochrome c oxidase activity determination, the heart membranes were incubated with DAB (1.4 mM) in the presence of cytochrome c (0.01%). DAB oxidation was measured every 2 min spectrophotometrically at 450 nM in a Multiskan FC microtiter plate reader (Thermo Scientific^®^, Waltham, MA, USA).

### 4.5. Determination of Superoxide Formation Mediated by Cannabis sativa Extracts

Bovine heart membranes were incubated in the presence of succinate (10 mM), NBT (0.05 mg/mL), and dUQ (10 µM) in the presence or absence of sodium azide (5 mM). Superoxide formation was measured every 2 min spectrophotometrically at 595 nM in a Multiskan FC microtiter plate reader (Thermo Scientific^®^, Waltham, MA, USA).

### 4.6. Data Analysis and Normalization

Data handling and analysis were carried out using Excel and GraphPad software (version 9.0). Results were expressed as means of independent data points ± S.E.M. Extracts-mediated cytochrome c oxidase inhibition is presented as a percentage of controls.

The relative concentration data (% composition values for each *Cannabis sativa* variety) were analyzed using STATGRAPHICS Centurion XIX (© 2022 Statgraphics Technologies, Inc., The Plains, Virginia, USA). Analysis of variance (ANOVA) and multiple range test (least significant difference) was used to detect significant differences (*p* < 0.5) between means. Multivariate cluster analysis (farthest neighbor method, housing block) was carried out to explore relationships between groups based on chemical composition and *Cannabis sativa* varieties. For the classification of the samples, the supervised models naive Bayes (NB), random forest (RF), and k-nearest neighbors (kNN) were applied using Orange software [40].

## Figures and Tables

**Table 1:** Content in CBD. CBDA. THC and THCA in the plant extracts expressed as μg/μg extract (mean ± SE).

Variety	CBD	CBDA	THC	THCA
A7	0.598 ± 0.012 a	0.057 ± 0.013 a	0.068 ± 0.003 a	0.010 ± 0.001 a
D2	0.638 ± 0.010 b	0.021 ± 0.005 b	0.070 ± 0.003 a	0.002 ± 0.001 b
N1	0.635 ± 0.014 ab	0.047 ± 0.004 ab	0.065 ± 0.010 ab	0.004 ± 0.001 bc
P0	0.630 ± 0.015 b	0.029 ± 0.007 ab	0.053 ± 0.005 b	0.008 ± 0.001 ac
T4	0.624 ± 0.004 ab	0.051 ± 0.010 a	0.072 ± 0.005 a	0.006 ± 0.001 c

Values within the same column followed by different lower-case letter are significantly different according to least significant difference (LSD) test (*p* < 0.05). Letters of each column reflect whether there are statistically significant differences in the relative content of that compound between varieties. Only the means marked with different letters are significantly different.

**Table 2:** *Cannabis sativa* varieties extraction yield expressed as % plant dry weight (average ± SE).

Variety	Average Yield %
A7	31.88 ± 2.03
D2	29.70 ± 0.85
N1	28.13 ± 0.74
P0	25.55 ± 2.46
T4	30.23 ± 1.32

## Data Availability

The data support the findings of this study are available from the corresponding author, Gabriel Barreda-Gómez, upon reasonable request.

## Supplementary Materials

The following supporting information can be downloaded at: https://www.mdpi.com/article/10.3390/ijms24021315/s1.
