# Supplementary material for: Screening System of Cannabis sativa Extracts Based on Their Mitochondrial Safety Profile Using Cytochrome c Oxidase Activity as a Biomarker
Source: Int J Mol Sci. 2023 Jan 10;24(2):1315. doi: 10.3390/ijms24021315 (PMC9864325; doi:10.3390/ijms24021315)
Supplement: Supplementary file 1 [file ijms-24-01315-s001.zip › ijms-2083164-supplementary.pdf]

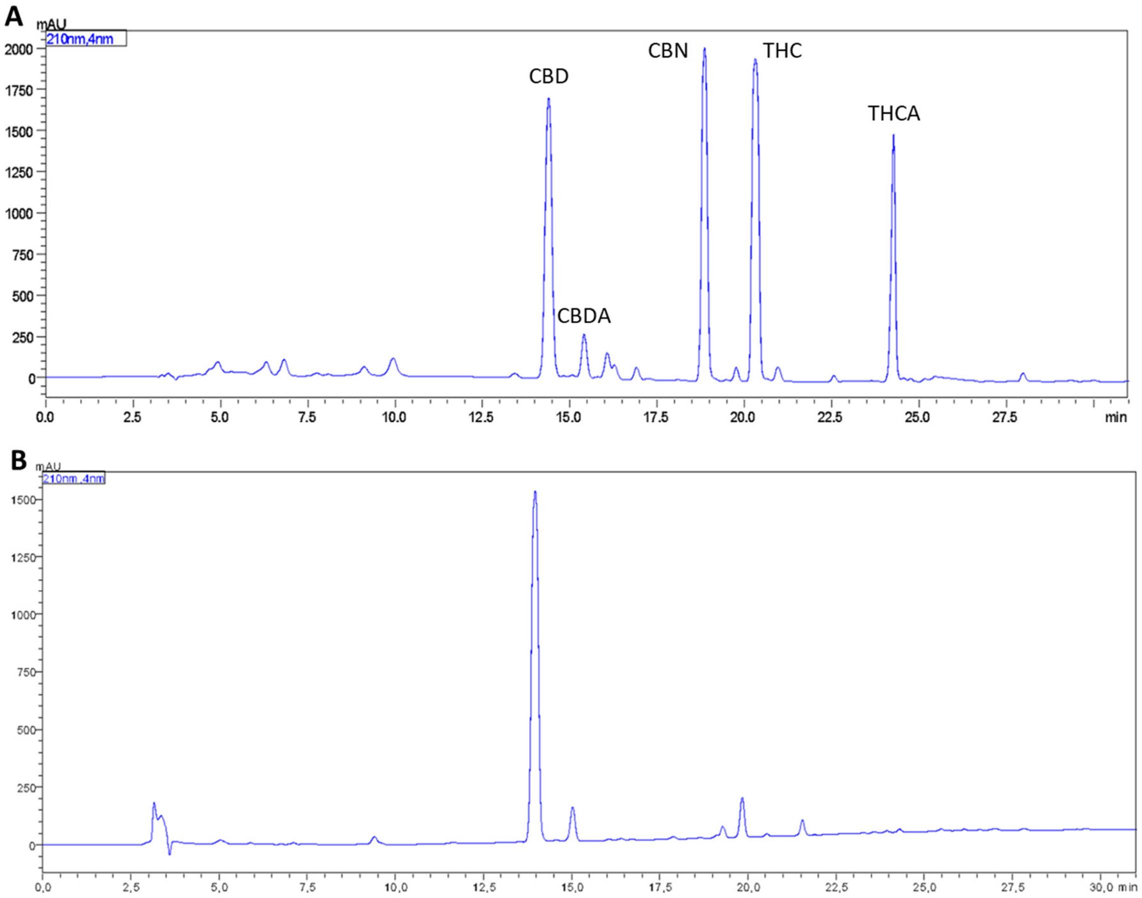

Figure S1. Chromatogram of standards (CBD, CBDA, CBN, THC, THCA) (**A**) and representative chromatogram of the sample N1-5 (**B**).

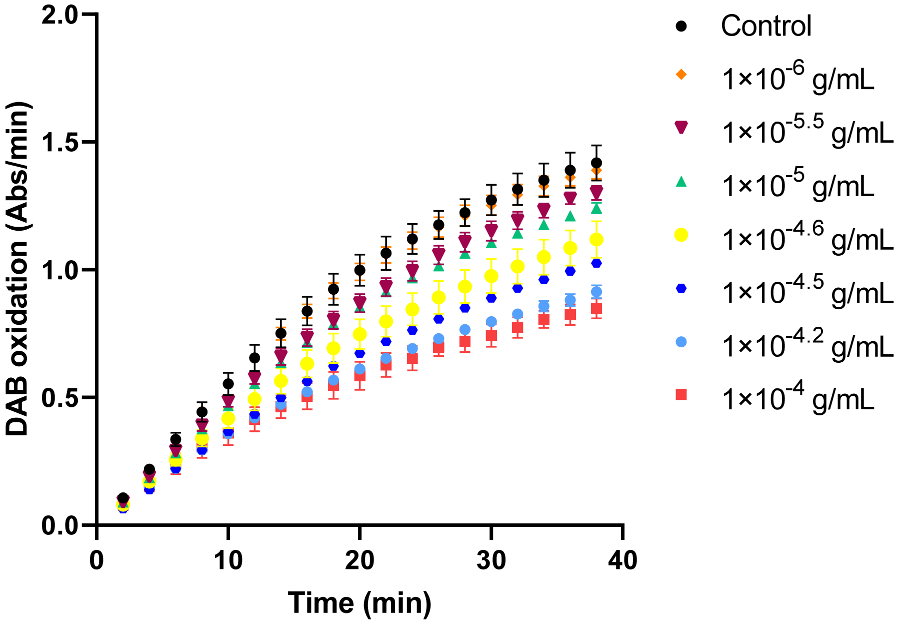

Figure S2. Representative graph of the DAB oxidation curves for the range of concentrations of the sample P0-3. Data are mean ± SEM values.

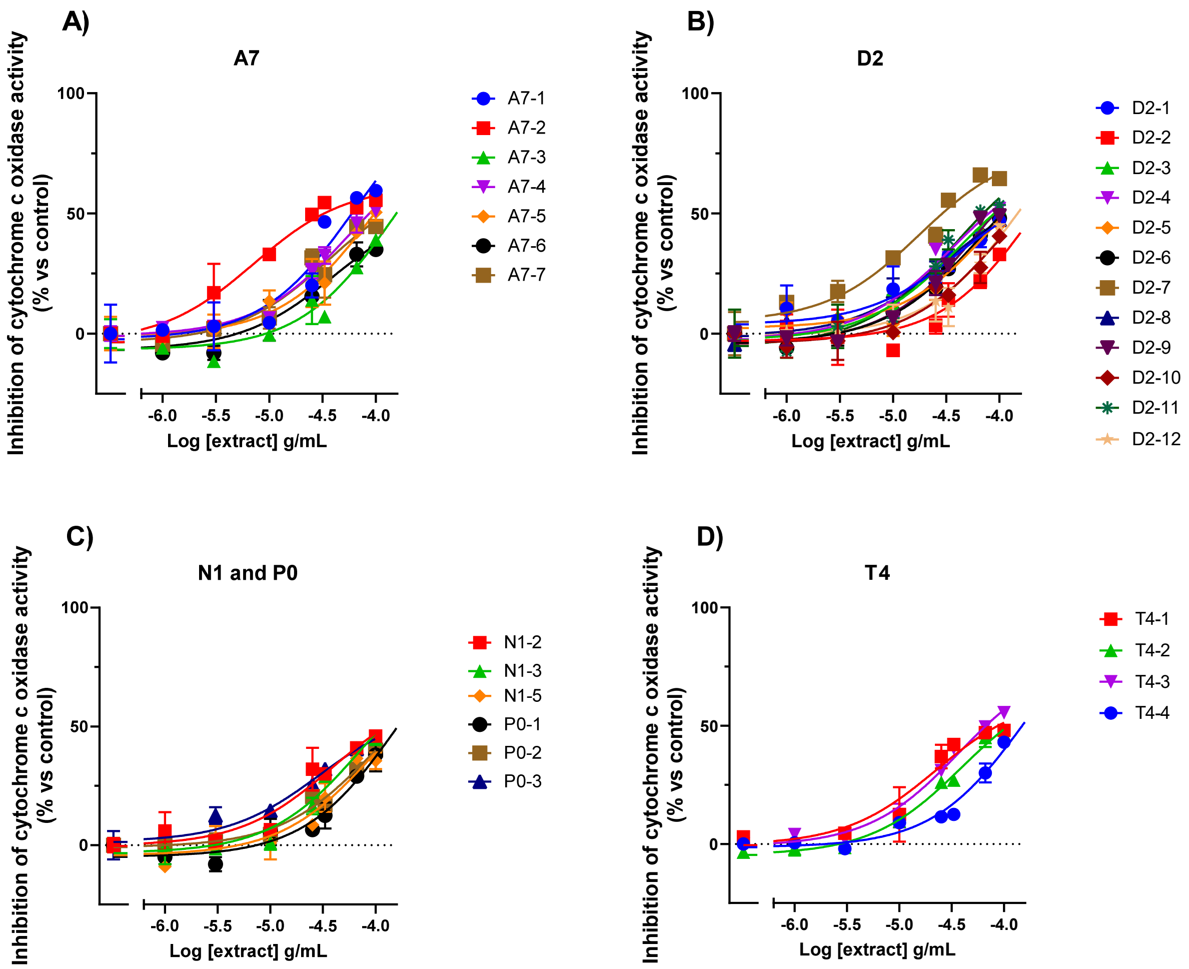

Figure S3. The concentration-effect curves of the variety A7 (A), variety D2 (B), varieties N1 and P0 (C), and variety T4 (D) on the CCO activity expressed as a percentage over the control. Data are mean ± SEM values.

Table S1. Chemical composition of the *Cannabis sativa* varieties analyzed (µg/µg extract) and Log EC50±SEM obtained in the CCO activity study. Samples within variety are clones obtained from the same mother plant.

Variety	Sample	Log EC50	±	SEM	CBD	CBDA	THC	THCA
**A7**	**A7-1**	-4.21	±	0.27	0.568	0.1061	0.075	0.011
	**A7-2**	-5.15	±	0.14	0.576	0.075	0.072	0.011
	**A7-3**				0.586	0.045	0.073	0.011
	**A7-4**	-4.26	±	0.13	0.594	0.033	0.066	0.01
	**A7-5**	-4.03	±	0.32	0.588	0.09	0.074	0.008
	**A7-6**	-4.40	±	0.21	0.614	0.023	0.054	0.008
	**A7-7**	-4.44	±	0.20	0.66	0.025	0.063	0.008
**D2**	**D2-1**	-4.38	±	0.28	0.65	0.057	0.063	0.001
	**D2-2**				0.601	0.01	0.0563	0.002
	**D2-3**	-4.38	±	0.12	0.628	0.015	0.0703	0
	**D2-4**	-4.44	±	0.11	0.597	0.01	0.074	0.003
	**D2-5**	-4.05	±	0.26	0.582	0.026	0.07	0.002
	**D2-6**	-4.21	±	0.13	0.696	0.018	0.062	0.004
	**D2-7**	-4.75	±	0.14	0.612	0.015	0.089	0.004
	**D2-8**	-4.50	±	0.15	0.655	0.019	0.074	0.001
	**D2-9**	-4.20	±	0.14	0.651	0.013	0.08	0.001
	**D2-10**	-4.01	±	0.32	0.66	0.012	0.064	0.007
	**D2-11**	-4.41	±	0.19	0.668	0.004	0.072	0.001
	**D2-12**				0.659	0.054	0.062	0.001
**N1**	**N1-2**	-4.42	±	0.22	0.66	0.04	0.071	0.0054
	**N1-3**	-4.13	±	0.22	0.61	0.055	0.0775	0.0045
	**N1-5**	-4.04	±	0.34	0.634	0.047	0.0462	0.0021
**P0**	**P0-1**				0.6	0.025	0.046	0.007
	**P0-2**	-4.04	±	0.17	0.649	0.0421	0.05	0.007
	**P0-3**	-4.54	±	0.17	0.642	0.0193	0.062	0.01
**T4**	**T4-1**	-4.55	±	0.27	0.63	0.024	0.086	0.008
	**T4-2**	-4.40	±	0.10	0.621	0.066	0.073	0.008
	**T4-3**	-4.43	±	0.10	0.631	0.068	0.063	0.003
	**T4-4**				0.613	0.045	0.067	0.005

Table S2. Extraction yield expressed as % plant dry weight

Variety	Sample	% Yield
A7	A7-1	29.78
	A7-2	31.57
	A7-3	41.04
	A7-4	36.40
	A7-5	31.98
	A7-6	26.08
	A7-7	26.34
D2	D2-1	25.30
	D2-2	40.18
	D2-3	26.54
	D2-4	32.74
	D2-5	32.31
	D2-6	29.88
	D2-7	28.94
	D2-8	29.32
	D2-9	28.58
	D2-10	36.32
	D2-11	27.90
	D2-12	29.43
N1	N1-1	29.23
	N1-2	26.72
	N1-3	28.45
	N1-4	29.82
	N1-5	23.20
P0	P0-1	26.05
	P0-2	29.54
	P0-3	21.05
T4	T4-1	26.70
	T4-2	32.20
	T4-3	29.71
	T4-4	32.32
